# Challenges and advantages of electronic prescribing system: a survey study and thematic analysis

**DOI:** 10.1186/s12913-024-11144-3

**Published:** 2024-05-30

**Authors:** Hamid Bouraghi, Behzad Imani, Abolfazl Saeedi, Ali Mohammadpour, Soheila Saeedi, Taleb Khodaveisi, Tooba Mehrabi

**Affiliations:** 1 Department of Health Information Technology, School of Allied Medical Sciences, Hamadan University of Medical Sciences, Shahid Fahmideh Blvd, Hamadan, Iran; 2 Department of Operating Room, School of Paramedicine, Hamadan University of Medical Sciences, Hamadan, Iran; 3 School of Medicine, Iran University of Medical Sciences, Tehran, Iran; 4 Health Information Management Department, Besat Hospital, Hamadan University of Medical Sciences, Hamadan, Iran

**Keywords:** Electronic prescribing system, Thematic analysis, Prescription, Challenges, Advantages

## Abstract

**Introduction:**

Electronic prescribing (e-prescribing) systems can bring many advantages and challenges. This system has been launched in Iran for more than two years. This study aimed to investigate the challenges and advantages of the e-prescribing system from the point of view of physicians.

**Methods:**

In this survey study and thematic analysis, which was conducted in 2023, a researcher-made questionnaire was created based on the literature review and opinions of the research team members and provided to the physician. Quantitative data were analyzed using SPSS software, and qualitative data were analyzed using ATLAS.ti software. Rank and point biserial, Kendall’s tau b, and Phi were used to investigate the correlation between variables.

**Results:**

Eighty-four physicians participated in this study, and 71.4% preferred to use paper-based prescribing. According to the results, 53.6%, 38.1%, and 8.3% of physicians had low, medium, and high overall satisfaction with this system, respectively. There was a statistically significant correlation between the sex and overall satisfaction with the e-prescribing system (*p*-value = 0.009) and the computer skill level and the prescribing methods (*P*-value = 0.042). Physicians face many challenges with this system, which can be divided into five main categories: technical, patient-related, healthcare providers-related, human resources, and architectural and design issues. Also, the main advantages of the e-prescribing system were process improvement, economic efficiency, and enhanced prescribing accuracy.

**Conclusion:**

The custodian and service provider organizations should upgrade the necessary information technology infrastructures, including hardware, software, and network infrastructures. Furthermore, it would be beneficial to incorporate the perspectives of end users in the system design process.

## Introduction

Medicine, a crucial commodity in healthcare due to its economic and strategic value, is a fundamental pillar in primary disease treatment. It constitutes significant health expenditures and budgets worldwide [1]. The prudent management of this valuable resource, through its appropriate prescription and usage, is essential. This is a key factor in ensuring the health security of communities [2]. Numerous studies indicate that errors in drug administration are prevalent. Although a significant proportion of these errors are preventable, they can leave serious complications for patients and even fatalities [3]. As the complexity of the drug prescribing process increases, resultant injuries and complications will likely escalate. Therefore, medication prescription is one of the main concerns and priorities of policymakers and trustees in the healthcare domain. In this regard, relentless endeavors are undertaken to enhance and optimize this process, and new supplementary solutions will be used as required. Employing electronic prescription (e-prescribing) systems as an alternative to manual prescription is a practical solution that can enhance and streamline this critical process [4].

In the traditional paper-based prescribing system, numerous issues arise, including illegible prescriptions, ambiguous orders, omissions, prescription forgery, and misidentification of patients. Studies indicate that these problems compromise patient safety and negatively impact the outcomes of drug treatments [5, 6]. E-prescribing emerges as an effective and definitive solution to the inefficiencies, susceptibility to fraud, and administrative burdens associated with paper-based prescribing systems [7]. E-prescribing extends beyond merely utilizing a computer for prescription writing and storage. This technology encompasses all stages of the prescription process, including patient identification, prescription registration, prescription modification, duplication and renewal of prescriptions, and the transfer of prescriptions among stakeholders, all facilitated through specialized software and internet platforms [8–10].

As an information system, the e-prescribing system can integrate with other organizational systems, such as electronic health records and pharmacy information systems, within healthcare centers like hospitals [11]. Through the implementation and utilization of such a system, it is possible to overcome the problems and constraints of the traditional prescribing system due to the complexity of medical care and the increase in the number of drugs, thereby benefiting from its potential advantages. Some of the benefits of an e-prescribing system include reducing healthcare costs for stakeholders (patients, healthcare providers, insurers, and policymakers), reducing common prescribing errors, improving medication outcomes, increasing patient safety, increasing the readability and accuracy of prescriptions, enhancing coordination among stakeholders involved in the drug therapy process, and supporting clinical decision-making at the time of drug administration [12–14].

Despite the potential benefits of e-prescribing systems in the healthcare industry and significant investments and efforts by stakeholders to support such systems, their usage and adoption remain low, resulting in the failure of numerous implemented projects [11, 12]. Given that e-prescribing systems are designed according to the specific needs and internal standards of each country, numerous studies have been conducted worldwide to investigate the benefits, challenges, the reasons for the failure and lack of acceptance of such systems [15, 16].

E-prescribing systems in countries like Denmark, the United States, Finland, Sweden, and the United Kingdom are commonly tested and implemented at state, local, or regional levels. These systems cover the entire or a significant portion of the prescribing process. Variations in healthcare and insurance systems across different countries lead to diverse approaches regarding e-prescribing and its evolution. Consequently, these countries exhibit distinct starting points, implementation procedures, and technical strategies. Moreover, e-prescribing systems and models vary not only across different countries but also within the same country [17]. While meticulously developed and successfully implemented in the United States of America, England, and Germany, this system has reached significant maturity and yielded substantial advantages for the health systems of these countries. However, in other nations, especially developing countries, e-prescribing still encounters significant challenges on its path to widespread acceptance and goal achievement [18–21].

Recognizing that the implementation of e-prescribing is a priority for the Iran Ministry of Health and Medical Education (MOH), the Iran Food and Drug Administration (IFDA) established a multi-stakeholder working group in 2015. This group, composed of medical informatics experts, aimed to develop recommendations for effective e-prescribing implementation [22]. In Iran, adopting e-prescribing in governmental and university hospitals has been proposed as a legal requirement since 2020. The Social Security Organization, a pioneering institution in this domain, has aligned with the implementation policies of this plan and has ceased issuing treatment booklets since early 2021 [23]. The Health Insurance Organization, as another government institution, independently developed and deployed its e-prescription system across all medical education centers affiliated with universities of medical sciences in Iran. Consequently, the two primary organizations (Social Security Organization and Health Insurance Organization) have successfully implemented the e-prescribing system. Their goals include efficient management of healthcare resources, reduction of common manual prescribing errors, and enhancement of patient safety [24].

In general, medical centers in Iran employ three distinct electronic prescription systems. “Electronic Prescription (EP)” and “Dinad” serve outpatients covered by the Social Security and Health Insurance Organization, while “Shafa” caters to all inpatients. For individuals without coverage from these insurances, physicians resort to paper prescriptions [25]. Electronic prescribing was not implemented simultaneously in all provinces of Iran. It was first used on a trial basis in a few provinces and then implemented throughout the country. Although these systems have provided significant benefits to their users in Iran, they have also encountered numerous challenges. Consequently, this comprehensive study was undertaken to explore both the advantages and obstacles associated with e-prescribing systems in Iran.

## Methods

This survey study and thematic analysis was conducted to examine the challenges and advantages of the e-prescribing system in Iran in 2023. This study was conducted in three main steps: literature review and questionnaire design, data collection, and data analysis.

### Literature review and questionnaire design

In the first step of this research, a questionnaire was designed based on the review of similar studies and the opinions of the research team members. To design the questionnaire, various databases, including PubMed, Google Scholar, and Scopus, were searched with related terms such as “electronic prescribing,” “electronic prescribing challenges,” and “electronic prescribing advantages.” Then, the most relevant articles retrieved from these databases were examined, and relevant data were extracted from these articles. Then, focus group sessions were held with the research team. The data extracted from the articles were presented in the sessions, and based on these data and the opinions of the research team, the questionnaire was finalized. This questionnaire had three sections: (1) demographic data (2), questions related to the advantages and challenges of e-prescribing, and (3) open-ended questions related to the challenges and advantages of the e-prescribing system. A five-point Likert scale from completely agree to completely disagree was used for the questions of the second part of the questionnaire. The face and content validity of the questionnaire was checked and confirmed with the cooperation of five experts in health information management, medical informatics, and information technology who were thoroughly familiar with prescribing systems. The content validity of the questionnaire was measured using the Content Validity Index (CVI) and Content Validity Ratio (CVR). To determine CVR, the experts were asked to classify each of the questions based on the three-point Likert scale as follows:

1. The question is necessary
2. The question is useful but not necessary
3. The question is not necessary

Then, the following formula was used to calculate CVR:

CVR = (Ne − N/2)/ (N/2), (N: total number of experts, Ne: the number of experts who have chosen the “necessary” option.).

Based on the Lawshe table for minimum values of CVR, items with CVR equal to or greater than 0.99 were kept. To calculate the CVI, the experts determined the degree of relevance of each question on a 4-point Likert scale from not relevant to completely relevant. The following formula was used to decide about the acceptance of each question:

CVI: The number of experts who chose options 3 and 4 / the total number of experts. It was decided to reject or accept each question as follows: < 0.7 = rejected, 0.7–0.79 = revised, > 0.79 = accepted. The reliability of the questionnaire was calculated using Cronbach’s alpha and Guttman coefficient. Values greater than 0.7, 0.5–0.7, and less than 0.5 indicate high, acceptable, and low reliability of the questionnaire, respectively.

The third part of the questionnaire included open-ended questions. Two following questions were placed at the end of the questionnaire and were asked to the physicians:

1. In your opinion, what other advantages does this electronic prescribing system have?
2. In your opinion, what other challenges does this electronic prescribing system have?

### Data collection

After the questionnaire was finalized, it was prepared in both paper and electronic formats. The electronic version of the questionnaire was prepared on the Porsline platform. For the survey, first, a list of physicians working in the teaching hospitals was prepared, and then we tried to get the contact numbers of the physicians as well. The questionnaire link was sent to physicians through the local social networks whose contact numbers were available, and physicians whose contact numbers were not available were referred to them in person. Many physicians refused to receive the questionnaire and answers due to lack of time. Two reminder messages were also sent to the doctors who had received the questionnaire link through social networks. In the face-to-face group, the doctors who did not have enough time to complete the questionnaire at that moment, the researcher provided the questionnaire to the physicians and coordinated with them to receive it at a later time. A total of 122 physicians agreed to participate in the study. It should be noted that to avoid missing data, it was mandatory to answer all the questions in the electronic questionnaire, and in the paper-based questionnaire, the researchers checked the questionnaire immediately, and if any fields were not completed, they asked the physicians to complete the incomplete items of the questionnaire again.

### Data analysis

Descriptive statistics including mean, standard deviation, frequency median, interquartile range and percentage were used for data analysis.

The relationship of “sex,” “specialty,” “physician’s computer skills,” “age,” and “duration” with “satisfaction” was investigated. Since “satisfaction” is a qualitative ordinal variable, the Rank-biserial index was used to examine the relationship between this variable and two-level nominal variables such as “gender” and “specialty.” Kendall’s tau b index was also used to examine the relationship between “satisfaction” (ordinal variable) with rank variables such as “physician’s computer skills” and continuous quantitative variables such as “age” and “duration.” To investigate the relationship between “willingness to use paper-based or e-prescribing” with “sex,” “specialty,” “physician’s computer skills,” “age,” and “duration,” Phi, Rank-biserial, and Point-biserial were used respectively. The *p*-values obtained from the chi-square test were also reported to check the presence or absence of a relationship between two variables. The type I error in this study was considered 5%. Data analysis was carried out using SPSS version 26.

The answers given by 84 physicians to two open-ended questions were typed in Word.

Thematic analysis was used to analyze the open-ended questions and identify themes within qualitative data. For thematic analysis, first, the answers typed in the Word were imported into the ATLAS.ti software, and then the pattern extraction process was carried out according to the following steps:

1. The imported text was read several times to get familiar with the data
2. After familiarizing with the data, initial coding was done
3. After coding, the extracted codes were checked and revised many times
4. Similar codes were merged and grouped, and subthemes were created
5. Finally, the sub-themes were reviewed and linked, and the main themes were created

## Results

The designed questionnaire was given to 122 physicians, of which 84 physicians completed the questionnaires (response rate: 68.85%). Demographic characteristics of physicians are given in Table 1. Most of the participants were general practitioners (56%) and women (53.6%). 91.7% of the physicians believed that they have medium and high computer skills and the average duration of using the e-prescribing system was 15.50 ± 8.798 months.

**Table 1:** Characteristics of physicians participating in the survey

Qualitative variable	Category	Frequency (percentage)
Sex	Male Female	39 (46.4) 45 (53.6)
Occupation	Specialist General practitioner	37 (44.0) 47 (56.0)
physician’s computer skills	Low Moderate High	7 (8.3) 51 (60.7) 26 (31.0)
**Quantitative variable**	**Scale**	**Mean (SD)**
Age	Year	32.86 ± 8.947
Duration of using the e-prescribing system	Month	15.50 ± 8.798

The results showed that the questionnaire had acceptable reliability (Cronbach’s alpha = 0.605, Guttman’s coefficient = 0.718). The mean (std. deviation), median and interquartile range of each question in the questionnaire are given in Table 2. The questions were categorized into two sections: advantages and challenges of the e-prescribing system. The total mean score of advantages for the e-prescribing system was 2.15 and this value for challenges of this system was 2.75. Out of the advantages of this technology, the highest mean score (2.79) was related to the “E-prescribing system has reduced the possibility of wrong drug delivery due to illegible prescriptions” and the lowest (1.24) was related to the “The e-prescribing system has led to improved physician performance”. The most important challenge that physicians had with the e-prescribing system was the insufficient bandwidth with an average of 3.49. Two other challenges mentioned by physicians about this system and received a high mean score (3.43) were the challenges related to lengthening the duration of each visit and increasing the waiting time of patients.

**Table 2:** Mean score and frequency of physicians’ attitudes towards e-prescribing system

#			Questions	Mean (Std. Deviation)	Median	Interquartile Range
1	**Advantages**	Improved workflow has resulted from e-prescribing system.		1.48 (1.217)	1	3
2		The availability of drug names has been enhanced by e-prescribing system.		2.37 (1.21)	3	2
3		The e-prescribing system has reduced the possibility of wrong drug delivery due to illegible prescriptions		2.79 (1.141)	3	2
4		E-prescribing system has led to a reduction in the number of drug prescriptions errors (such as drug names, drug doses, and treatment courses).		2.24 (1.257)	3	2
5		The e-prescribing system has led to improved physician performance.		1.24 (1.06)	1	2
6		Pre-prepared prescriptions for common diseases have been created by e-prescribing.		2.7 (1.084)	3	0
7		The number of phone calls between doctors and pharmacies has been cut down by e-prescribing to ensure the correctness of the prescribed medication.		2.23 (1.264)	3	2
Total mean score of advantages				**2.15**		
1	**Challenges**	E-prescribing has increased the number of errors in drug prescribing.		1.8 (1.117)	1.5	2
2		E-prescribing has increased the confusion among doctors.		2.77 (1.01)	3	2
3		E-prescribing has increased the waiting times for patients.		3.43 (0.826)	4	1
4		E-prescribing has reduced patient satisfaction (due to forgetting the national code, increasing the waiting time, etc.).		2.99 (1.058)	3	2
5		E-prescribing has reduced the possibility of viewing drug prescription records (in paper prescribing it was possible to view previously prescribed drugs).		2.61 (1.182)	3	3
6		Doctors have faced challenges with e-prescribing due to insufficient bandwidth.		3.49 (0.814)	4	1
7		E-prescribing has increased the time needed to prescribe medicine due to typographical errors, system failures, etc.		3.43 (0.796)	4	1
8		E-prescribing has resulted in the prescription of drugs based on the pharmaceutical company.		2.43 (1.021)	2	1
9		E-prescribing has necessitated doctors to remember drug codes.		2.15 (1.07)	2	2
10		E-prescribing has led to doctors spending a lot of time for searching drug codes.		2.98 (1.075)	3	2
11		E-prescribing and lack of access to computer systems by some doctors have increased the prescription of drugs without insurance.		2.9 (0.952)	3	2
12		E-prescribing has caused long waiting lines for patients.		3.27 (0.923)	3.5	1
13		Doctors encounter technical complexity when working with e-prescribing systems.		2.21 (1.183)	2.5	2
14		The e-prescribing system has led to an increase in the incorrect retrieval of patients’ names, which has resulted in prescribing drugs to another patient.		1.79 (1.087)	2	1
15		Physicians are facing an excessive workload due to e-prescribing.		2.92 (1.1)	3	2
16		E-prescribing has led to the limitation of doctors in prescribing some drugs.		2.86 (0.996)	3	2
Total mean score of challenges				**2.75**		

The results of investigating the correlation between the duration of e-prescribing system use, age, sex, specialty, and the physician’s computer skills with the overall satisfaction with the e-prescribing system are reported in Table 3. According to the results, 45 (53.6%), 32 (38.1%), and 7 (8.3%) physicians had low, medium and high overall satisfaction with this system, respectively. There was a statistically significant correlation between the sex and overall satisfaction with the e-prescribing system (*p*-value = 0.009).

**Table 3:** Correlation between independent variables and the level of satisfaction with e-prescribing system

		Satisfaction			Coefficient value	*P*-value
		Low	Moderate	High		
Variable	Category	Frequency (%)	Frequency (%)	Frequency (%)		
Sex	Female	29(64.4)	15(46.9)	1(14.3)	0.283^*^	0.009
	Male	16(35.6)	17(53.1)	6(85.7)		
Specialty	Specialist	21(46.7)	15(46.9)	1(14.3)	0.122^*^	0.271
	GP	24(53.3)	17(53.1)	6(85.7)		
physician’s computer skills	Low	3(6.7)	4(12.5)	0(0.0)	0.109^ǂ^	0.306
	Moderate	30(66.7)	19(59.4)	2(28.6)		
	High	12(26.7)	9(28.1)	5(71.4)		
Total	-	45(53.6)	32(38.1)	7(8.3)	-	-
**Variable**	**Scale**	**Mean (SD)**	**Mean (SD)**	**Mean (SD)**	**Coefficient value**	***P*** **-value**
Age	Year	33.31(9.177)	31.94(8.504)	34.14(10.399)	-0.029^ǂ^	0.753
Duration	Month	16.91(7.701)	13.84(10.119)	14.00(8.641)	-0.144^ǂ^	0.100

The results of the correlation between duration, age, sex, specialty, and the physician’s computer skills with the willingness to use paper-based prescribing or the e-prescribing system are reported in Table 4. According to the results, 60 (71.4%) and 24 (28.6%) physicians preferred to use paper-based and e-prescribing respectively. There was a statistically significant correlation between the computer skill level and the prescribing methods (*P*-value = 0.042).

**Table 4:** Correlation between independent variables and the willingness to use paper-based or e-prescribing

		Willingness to use paper-based or e-prescribing		Coefficient value	*P*-value
		Paper-based	Electronic		
Variable	Category	Freq (%)	Freq (%)		
Sex	Female	36(60.0)	9(37.5)	0.204^*^	0.090
	Male	24(40.0)	15(62.5)		
Specialty	Specialist	30(50.0)	7(29.2)	0.190^*^	0.094
	GP	30(50.0)	17(70.8)		
physician’s computer skills	Low	3(5.0)	4(16.7)	0.260^**^	0.042
	Moderate	41(68.3)	10(41.7)		
	High	16(26.7)	10(41.7)		
Total	-	60 (71.4)	24 (28.6)	-	-
**Variable**	**Scale**	**Mean (SD)**	**Mean (SD)**	**Coefficient value**	***P*** **-value**
Age	Year	33.88(9.602)	30.29(6.537)	-0.182^ǂ^	0.097
Duration	Month	15.80(8.914)	14.75(8.644)	-0.054^ǂ^	0.624

^*^: Phi; ^**^: Rank-biserial; ^ǂ^: Point-biserial; SD: Standard Deviation.

**Fig. 1:**
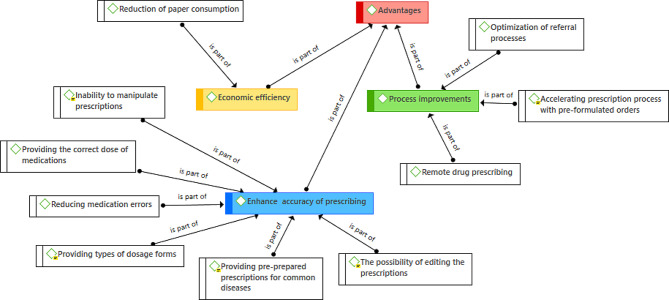
Thematic map of concepts extracted from qualitative data related to the advantages of the e-prescribing system

The themes and sub-themes extracted from the question related to the advantages of the e-prescribing system are shown in Fig. 1. The main themes of the e-prescribing system’s advantages were the following:

1. Process improvement
2. Economic efficiency
3. Enhance the accuracy of prescribing

These three themes included a total of 10 sub-themes.

Among the advantages noted for electronic prescribing, the possibility of editing prescriptions, providing different dosages of drugs, and the impossibility of manipulating prescriptions by patients or other people were mentioned more than other advantages. Also another mentioned advantage was the possibility of providing pre-prepared prescriptions for common diseases, which led to the acceleration of prescribing for these diseases.

**Fig. 2:**
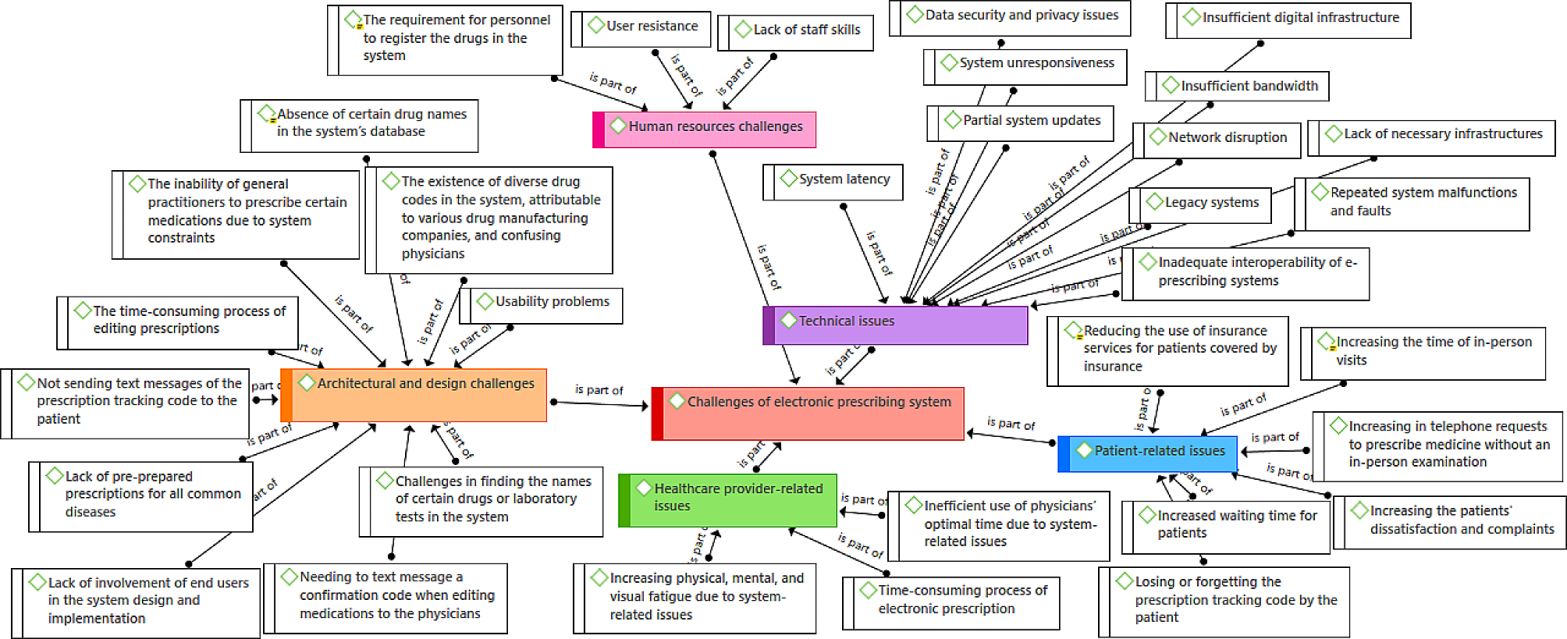
Thematic map of concepts related to the challenges of the e-prescribing system

Concepts related to the challenges of the e-prescribing system were categorized into five main themes as follows (Fig. 2):

1. Technical issues
2. Patient-related issues
3. Healthcare providers-related issues
4. Human resources challenges
5. Architectural and design issues

These five themes included more than 30 sub-themes.

Many challenges for electronic prescribing were mentioned in the form of given themes. One of the most important challenges mentioned by many physicians was various technical problems including network disconnection. Also, another big challenge that caused the dissatisfaction of the patients was the lack of skill of many physicians in working with computer systems, which led to the low speed of typing the drugs in the system and as a result, increased the duration of the patients’ visits. Also, many physicians did not have computer systems in their clinics, which led to the lack of electronic prescriptions and, as a result, the lack of use of insurance services for patients. Also, considering that many physicians are used to the paper prescription method, they were not willing to accept the changes and resisted these changes, as a result, they needed personnel to register the prescriptions.

## Discussion

E-prescribing systems have many advantages, but they also pose certain challenges. These systems can enhance medication safety by reducing prescription errors caused by illegible handwriting or oral miscommunication. They can also improve efficiency by streamlining the prescription process, reducing the time spent on phone calls and faxes between healthcare providers and pharmacies. Furthermore, e-prescribing can provide clinicians with up-to-date information about patients’ medications and allergies, thereby improving patient care.

Although e-prescribing systems have many advantages, their implementation is not without any challenges. These include the costs associated with system implementation and maintenance, issues related to system interoperability, and the necessity for user training and technical support. Moreover, while these systems can mitigate traditional medication errors, they may also introduce new types of errors, such as those caused by user interface design or software glitches. Maximizing the benefits and minimizing the challenges associated with e-prescribing systems requires meticulous system design, comprehensive user training, and continuous system evaluation.

As demonstrated in the results section, the e-prescribing system’s mean overall benefit score was 2.15. This score suggests a moderate level of perceived benefits. It implies that while certain advantages are acknowledged, the system still needs to be improved to enhance user satisfaction and the perception of benefits. In this context, among the factors associated with the system’s benefits from the users’ perspective, the statements “Improved workflow has resulted from e-prescribing” and “The e-prescribing system has led to improved physician performance” received average scores of 1.48 and 1.24, respectively. These relatively low scores suggest that respondents of the survey or study largely disagree that the electronic system has enhanced their workflow or improved their performance. Several studies [11, 12, 26, 27] have demonstrated that users do not concur that the use of prescribing systems leads to workflow improvement or performance enhancement. There are multiple possible reasons for this, including:

Usability issues: The e-prescribing system might not be user-friendly or intuitive, leading to difficulties in adoption among healthcare professionals.

Training and support: There might be a lack of adequate training and support for the users, making it challenging for them to adapt to the new system.

System limitations: The system might not be flexible enough to accommodate the diverse needs of different healthcare settings, leading to workflow inefficiencies.

Resistance to change: Healthcare professionals, like any other group, might resist changes to established routines. This resistance could affect their perception of the system’s benefits.

Among the challenges identified in the use of e-prescribing systems, the statement “Doctors have faced challenges with e-prescribing due to insufficient bandwidth” received the highest score of 3.49. According to this relatively high score, the survey or study respondents strongly agree that insufficient bandwidth has been a significant obstacle to the use of e-prescribing. This issue results in prolonged patient waiting times, leading to extended queues and a decrease in physician productivity. There are multiple factors that can cause insufficient bandwidth, such as:

Network Infrastructure: In areas with poor network infrastructure, insufficient bandwidth can significantly slow down the operation of e-prescribing systems, making it difficult for doctors to use them effectively.

System Requirements: To function optimally, e-prescribing systems may need a certain level of bandwidth. System lags or downtime could result if the available bandwidth is below this level.

Data Transfer: E-prescribing systems often need to transfer large amounts of data, including patient records, prescriptions, and other related information. Insufficient bandwidth can slow down this data transfer, affecting the system’s efficiency.

Real-time Updates: Many e-prescribing systems provide real-time updates to ensure that all users have the most current information. If there is not enough bandwidth, these updates can be delayed, resulting in potential errors or miscommunications.

Generally, as indicated by various studies [28–30], the implementation of e-prescribing systems requires robust hardware, sophisticated software, and a reliable network infrastructure. These elements are integral to the successful deployment and operation of such systems. According to this study, the hardware, software, and network infrastructure in Iran are not suitable for the implementation of e-prescribing systems. This inadequacy has caused increased challenges and dissatisfaction among users. Furthermore, our evaluation of physicians’ overall satisfaction with the e-prescribing system revealed that the majority, 45 (53.6%), had low satisfaction. Conversely, only a small proportion, 7 (8.3%), reported high satisfaction. Subsequently, the e-prescribing system is not widely accepted by users, with the majority (71.4%) favoring paper-based prescribing. Many other studies have indicated higher levels of user satisfaction and a greater willingness to accept and use e-prescribing systems, contrary to our study’s findings [31–34]. The low level of satisfaction and users’ reluctance to adopt the e-prescribing system can be attributed to various challenges and problems identified by them. Users have been greatly impacted by these issues, which range from technical difficulties to system design and architecture issues, resulting in dissatisfaction, diminished motivation, and resistance towards the system.

## Conclusion

Although e-prescribing systems represent a novel and transformative approach in healthcare, they offer numerous benefits, including improved efficiency, reduced medication errors, and enhanced patient safety. However, our study highlights the presence of significant challenges, such as technical issues and problems related to system design and architecture, which result in low user satisfaction and hinder system adoption. The custodian and service provider organizations should upgrade the necessary information technology infrastructures, including hardware, software, and network infrastructures, to address the technical challenges. Furthermore, given that the design and architectural issues of the e-prescribing systems have resulted in user dissatisfaction and diminished motivation to use the system, identifying and addressing these problems and shortcomings in future updates is recommended. Moreover, it is important to take into account the end users’ perspectives during the system design process.

## Data Availability

All data generated or analyzed during this study are included within this article.
